# Ingredients from *Litsea garrettii* as Potential Preventive Agents against Oxidative Insult and Inflammatory Response

**DOI:** 10.1155/2018/7616852

**Published:** 2018-03-20

**Authors:** Yan-Ru Li, Guo-Hui Li, Lin Sun, Lin Li, Yue Liu, De-Gang Kong, Shu-Qi Wang, Dong-Mei Ren, Xiao-Ning Wang, Hong-Xiang Lou, Tao Shen

**Affiliations:** ^1^Key Lab of Chemical Biology (MOE), School of Pharmaceutical Sciences, Shandong University, 44 Wenhua Xi Road, Jinan 250012, China; ^2^Department of Pharmacy, Jinan Maternity and Child Care Hospital, Jianguo Xiaojingsan Road, Jinan 250000, China

## Abstract

Oxidative stress and inflammation undoubtedly contribute to the pathogenesis of many human diseases. The nuclear transcription factor erythroid 2-related factor (Nrf2) and the nuclear factor *κ*B (NF-*κ*B) play central roles in regulation of oxidative stress and inflammation and thus are targets for developing agents against oxidative stress- and inflammation-related diseases. Our previous study indicated that the EtOH extract of *Litsea garrettii* protected human bronchial epithelial cells against oxidative insult via the activation of Nrf2. In the present study, a systemic phytochemical investigation of *L. garrettii* led to the isolation of twenty-one chemical ingredients, which were further evaluated for their inhibitions on oxidative stress and inflammation using NAD(P)H:quinone reductase (QR) assay and nitric oxide (NO) production assay. Of these ingredients, 3-methoxy-5-pentyl-phenol (MPP, 5) was identified as an Nrf2 activator and an NF-*κ*B inhibitor. Further studies demonstrated the following: (i) MPP upregulated the protein levels of Nrf2, NAD(P)H:quinone oxidoreductase 1 (NQO1), and glutamate-cysteine ligase regulatory subunit (GCLM); enhanced the nuclear translocation and stabilization of Nrf2; and inhibited arsenic [As(III)]-induced oxidative insult in normal human lung epithelial Beas-2B cells. And (ii) MPP suppressed the nuclear translocation of NF-*κ*B p65 subunit; inhibited the lipopolysaccharide- (LPS-) stimulated increases of NF-*κ*B p65 subunit, COX-2, iNOS, TNF-*α*, and IL-1*β*; and blocked the LPS-induced biodegrade of I*κ*B-*α* in RAW 264.7 murine macrophages. Taken together, MPP displayed potential preventive effects against inflammation- and oxidative stress-related diseases.

## 1. Introduction

Exposure of cells and tissues to the environmental oxidants and toxicants (e.g., heavy metals, xenobiotics, pathogen, free radicals, drugs, and ionizing radiation) gives rise to oxidative stress, which is characterized by the overproduction of reactive oxygen species (ROS) [1]. Excess endogenous ROS activates redox-sensitive transcription factors such as the nuclear factor *κ*B (NF-*κ*B), which regulates inflammatory mediator gene expression to bring out inflammation [2]. Subsequently, the increased releases of inflammatory mediators promote the production of endogenous ROS to enhance oxidative stress in multiple cell types. Therefore, environmental oxidants and toxicants activate a positive feedback loop between oxidative stress and inflammation, which initiates and amplifies the pathophysiology of many human diseases, including cancer, neurodegenerative diseases, cardiovascular disease, diabetes mellitus, and chronic obstructive pulmonary disease (COPD) [3–7]. Based on these observations, dual inhibitions of oxidative stress and inflammation are regarded to be an efficient strategy to block the pathogenesis of these diseases.

The nuclear transcription factor erythroid 2-related factor- (Nrf2-) regulated defensive response is the main defense system of maintaining the intracellular redox homeostasis [8]. Under basal conditions, Nrf2 is sequestered by Keap1 in the cytosol and maintained at a low level through Keap1-mediated ubiquitylation and subsequent 26S proteasome-regulated degradation. When the intracellular redox imbalance emerges, Nrf2 escapes from Keap1, translocates into the nucleus, binds to the antioxidant response element (ARE) located in the promoter region of cytoprotective genes, and upregulates their transcription. These cytoprotective genes include intracellular redox-balancing proteins [e.g., glutamate-cysteine ligase regulatory subunit (GCLM)] and phase II detoxifying enzymes [e.g., NAD(P)H:quinone oxidoreductase 1 (NQO1)], which are able to maintain the cellular redox capacity and promote excretion of toxicants [9–11].

NF-*κ*B is a redox-sensitive transcription factor and plays a critical role in the regulation of genes in response to inflammatory stimuli [12]. Under normal conditions, NF-*κ*B is bound to inhibitors of *κ*B (I*κ*B) proteins in the cytoplasm. When cells were exposed to stimuli, I*κ*B was phosphorylated by IKK to release NF-*κ*B into the nucleus and drive the expression of inflammation-related genes [13]. These inflammatory genes include proinflammatory cytokines [e.g., tumor necrosis factor *α* (TNF-*α*) and interleukin-1*β* (IL-1*β*)], chemokines, adhesion molecules (e.g., E-selectin), and inflammation-related proteins [e.g., cyclooxygenase-2 (COX-2) and inducible nitric oxide synthase (iNOS)] [14].

On the basis of biological functions of Nrf2 and NF-*κ*B, characterization of molecules that activate Nrf2-mediated defensive responses and inhibit NF-*κ*B-regulated inflammatory response is a meaningful strategy for discovering potential preventive agents against oxidative stress- and inflammation-related diseases. Discoveries of molecules with therapeutic effects against oxidative stress- and/or inflammation-related diseases are our continuous projects [15–17]. In our previous study, the EtOH extract of *Litsea garrettii* Gamble [synonym *L. martabanica* (Kurz) Hook. f.], a plant distributed in Thailand, Myanmar, and the south of China [18], protected human bronchial epithelial (HBE) cells against H_2_O_2_-induced oxidative insults in an Nrf2-dependent manner [16]. However, no investigation on its phytochemical aspects has been reported, and accordingly, the chemical composition of this plant remains unknown.

In the present study, we characterized the chemical composition of *L. garrettii* and tested their inhibitory effects on oxidative stress and inflammatory response using NAD(P)H:quinone reductase (QR) inducing assay and nitric oxide (NO) production assay. 3-Methoxy-5-pentyl-phenol (MPP, **5**) was identified to be an activator of Nrf2 signaling pathway and an inhibitor of NF-*κ*B signaling pathway for the first time. Our results indicated that MPP protected human lung epithelial cells against arsenic [As(III)]-induced oxidative insult in Beas-2B cells and prevented LPS-induced inflammatory response in RAW 264.7 macrophages. Collectively, MPP demonstrated potential preventive effects against inflammation- and oxidative stress-related diseases.

## 2. Materials and Methods

### 2.1. Chemicals

Sulforaphane (SF), menadione, digitonin, As(III), and flavin adenine dinucleotide were purchased from Sigma-Aldrich (MO, USA). Didox was purchased from MedChemExpress (Monmouth Junction, NJ, USA). Cycloheximide (CHX), 4′,6-diamidino-2-phenylindole (DAPI), 3-(4,5-dimethylthiazol-2-yl)-2,5-diphenyltetrazoliumbromide (MTT), bromophenol blue, glycerol, and bovine serum albumin (BSA) were purchased from Genview (TX, USA). *β*-Mercaptoethanol was obtained from Dingguo (Beijing, China). Eagle's minimal essential medium (MEM) and RPMI 1640 were acquired from Gibco (CA, USA). Fetal bovine serum (FBS) was purchased from Gemini Bio Products (CA, USA). Glucose-6-phosphate and L-glutamine were obtained from Solarbio (Beijing, China). Glucose-6-phosphate dehydrogenase and nicotinamide adenine dinucleotide phosphate (NADP) were obtained from Regal (Shanghai, China). Solvents used for extraction and isolation were of analytical grade and obtained from Tianjin Fuyu Fine Chemical Company (Tianjin, China). Naphthylethylenediamine and sulfanilamide were obtained from Sinopharm Chemical Reagent Co. Ltd. (China).

### 2.2. General Experimental Procedures

^1^H and ^13^C NMR spectra were acquired on a Bruker Avance 600 or a Bruker Avance 400 instrument. A LTQ-Orbitrap XL instrument was used for the determination of high-resolution ESI-MS mass spectra. Semipreparative HPLC using a Shimadzu SPD-20A instrument (Shimadzu, Japan) equipped with an YMC-Pack ODS-A column (250 × 10 mm, 5 *μ*m) was used for sample isolation. Silica gel (200–300 mesh, Haiyang Co., Qingdao, China) and Sephadex LH-20 gel (Amersham Biosciences) were adopted for column chromatography. TLC analysis was performed on precoated silica GF254 plates (Haiyang Co., Qingdao, China).

### 2.3. Plant Material

The aerial parts of *L. garrettii* Gamble were collected from Xishuangbanna, Yunnan Province, China, in September 2011 and identified by Professor Lan Xiang, School of Pharmaceutical Sciences, Shandong University. A voucher specimen (no. XSBN2011-ZK-08) was deposited at the Laboratory of Pharmacognosy, School of Pharmaceutical Sciences, Shandong University.

### 2.4. Extraction and Isolation

The aerial parts of *L. garrettii* (5.38 kg) were extracted with 95% EtOH (10 L × 3). The dried EtOH extract (210 g) was dissolved in water and fractionated successively with petroleum ether, EtOAc, and *n*-BuOH. The petroleum ether-soluble extract (86.6 g) was separated by silica gel column chromatography (CC) and eluted with a gradient of PE-EtOAc (100 : 0→40 : 60) to give fourteen fractions (Frs. P1–P14). **1** (1.0 g) was crystallized from fr. P6. Fr. P7 was subjected to Sephadex LH-20 CC and semipreparative HPLC to furnish **2** (10.1 mg), **3** (1.8 mg), and **4** (10.6 mg). **5** (7.5 mg) was isolated from fr. P8 by Sephadex LH-20 CC and semipreparative HPLC. Fr. P9 was separated over a Sephadex LH-20 CC to yield 10 subfractions (Frs. P9a-j). **6** (100.5 mg) and **7** (4.3 mg) were purified from fr. P9e and fr. P9j, respectively. **8** (12.7 mg), **9** (18.6 mg), and **10** (25.3 mg) were purified from frs. P11, P12, and P14, respectively, by combined applications of Sephadex LH-20 CC and semipreparative HPLC.

The EtOAc-soluble fraction (78.8 g) was fractionated by silica gel CC using a gradient of petroleum ether-EtOAc (100 : 0→0 : 100), to give fifteen fractions (Frs. E1–15). **11** (6.4 mg) was obtained from fr. E8 by Sephadex LH-20 CC. Fr. E9 was purified by Sephadex LH-20 CC to yield seven subfractions (Frs. E9a-g). **12** (28.5 mg) and **13** (6.8 mg) were isolated from fr. E9e by semipreparative HPLC. Fr. E9f was subjected to semipreparative HPLC to yield **14** (34.2 mg). Fr. E10 was separated over Sephadex LH-20 CC to afford eight subfractions (Frs. E10a-h). Fr. E10e was further fractionated by semipreparative HPLC to yield **15** (8.1 mg). **16** (4.9 mg) was isolated from fr. E10h by semipreparative HPLC. A Sephadex LH-20 CC was adopted for the fraction of fr. E11 to furnish nine subfractions (Frs. E11a-i). **17** (43.9 mg) was purified from fr. E11d by semipreparative HPLC. **18** (4.8 mg) was precipitated from fr. E11g. Fr. E14 was chromatographed on a Sephadex LH-20 to afford three subfractions (Frs. E14a-c). **19** (50.5 mg) and **20** (3.8 mg) were crystallized from frs. E14b and 14c, respectively. Fr. E15 was purified by Sephadex LH-20 CC to give **21** (20.5 mg).

### 2.5. Cell Culture

Hepa 1c1c7 murine hepatoma cells (American Type Culture Collection, ATCC, USA) were maintained in Eagle's minimal essential medium (MEM, Gibco, USA) supplemented with 10% fetal bovine serum (FBS, Gemini Bio Products, USA), 100 units/mL penicillin, and 100 *μ*g/mL streptomycin. RAW 264.7 murine macrophages (ATCC, USA) and normal human lung epithelial Beas-2B (ATCC, USA) were cultured in 1640 medium (Gibco, USA) supplemented with 10% FBS, 100 units/mL penicillin, and 100 *μ*g/mL streptomycin. Cells were incubated at 37°C in humidified under 5% CO_2_.

### 2.6. NAD(P)H:Quinone Reductase (QR) Assay

Hepa 1c1c7 cells were seeded in 96-well plates (1 × 10^4^ cells/well) and exposed to the indicated doses of tested ingredients for 24 h. After the medium was decanted, the cell was treated with 30 *μ*L of lysing solution [0.8% digitonin and 2 mM EDTA solution (pH 7.8)] for 15 min at 37°C. Then, the complete reaction mixture (170 *μ*L) was prepared by mixing 15 mg bovine serum albumin, 6 mg MTT, 150 *μ*L 1.5% Tween 20, 1 mL 0.5 M Tris-HCl, 15 *μ*L 7.5 mM flavin adenine dinucleotide, 150 *μ*L 150 mM glucose-6-phosphate, 6 *μ*L 10 units/*μ*L glucose-6-phosphate dehydrogenase, 15 *μ*L 50 mM NADP, 20 *μ*L 50 mM menadione, and 18.4 mL H_2_O and added into the cell lysates for 170 *μ*L/well. After incubation for 4 min, the absorbance was measured at 630 nm on Model 680 plate reader (Bio-Rad, CA, USA).

### 2.7. Nitric Oxide (NO) Production Assay

RAW 264.7 cells (8 × 10^4^ cells/well) were seeded into 96-well plates and treated with LPS (1 *μ*g/mL) in the absence or presence of tested ingredients for 24 h. Then, 100 *μ*L of supernatant was mixed with equal volume of Griess reagent (0.1% naphthylethylenediamine and 1% sulfanilamide in 5% H_3_PO_4_ solution) in a new 96-well plate. After incubation at room temperature for 15 min, the absorbance was measured at 570 nm on Model 680 plate reader, and the amount of NO was assessed by a NaNO_2_ standard curve.

### 2.8. Cell Viability Assay

Following the NO production assay, we used a MTT assay to measure cytotoxicity of tested ingredients against RAW 264.7 cells. Briefly, after removing 100 *μ*L supernatant for detection of the NO concentration as described in Section 2.6, we added 100 *μ*L of 1640 media containing 0.4% MTT to each well and incubated them at 37°C for 3 h. Then, the supernatant was discarded carefully, and cells containing reduced MTT were dissolved in 100 *μ*L DMSO. After a brief period of shaking, the absorbance was measured at 570 nm by a Model 680 plate reader. MTT assay has also been used to measure the antiproliferative effect of MPP. Cells (2 × 10^4^ cells/well) were seeded in a 96-well plate and treated with indicated doses of MPP. After culturing for the indicated time, the absorbance was measured 570 nm.

### 2.9. Immunofluorescence

Beas-2B cells (4.0 × 10^4^ cells/well) or RAW 264.7 cells (6.0 × 10^5^ cells/well) were seeded in a 12-well plate, which have been preplaced by cell climbing pieces at the bottom, and were treated with MPP for indicated times or pretreated with indicated doses of MPP for 1 h followed by treatment with LPS (1 *μ*g/mL) for another 1 h. After being fixed with methanol/acetone (1 : 1), cells were incubated with primary rabbit anti-Nrf2 antibody or primary antirabbit p65 antibody for 3 h. After being washed with PBS for 3 times, the cell climbing pieces were incubated with red fluorescent Alexa Fluor 594 (Proteintech Group, USA) for 1 h and followed by nuclei labeling with 4′,6-diamidino-2-phenylindole (DAPI) (Genview, USA) for 10 min. The fluorescence signals were imaged using Olympus BX53 fluorescence microscope coupled to Olympus DP73 digital camera (Tokyo, Japan).

### 2.10. Luciferase Reporter Gene Assay

ARE-luciferase reporter gene assay and NF-*κ*B luciferase reporter gene assay were applied to measure the transcriptional activities of Nrf2 and NF-*κ*B in response to the MPP treatment, respectively. Beas-2B cells or RAW 264.7 cells were transfected with ARE-firefly luciferase or NF-*κ*B firefly luciferase, and a TK-Renilla luciferase plasmid using Lipofectamine 2000 reagent (Invitrogen, USA). Cells were exposed to MPP for 16 h or pretreated with different concentrations of MPP for 1 h followed by stimulation with LPS (1 *μ*g/mL) for another 16 h. After that, the firefly and Renilla luciferase activities were measured using the Dual-Luciferase Reporter gene assay system (Promega, USA).

### 2.11. Immunoblot Analysis and Protein Half-Life Measurement

Antibodies for p65, I*κ*B-*α*, NQO1, and Lamin B were purchased from Santa Cruz Biotechnology (USA). Antibodies for Nrf2, Keap1, COX-2, iNOS, GCLM, PCNA, and *β*-actin were purchased from Proteintech Group (USA). Cells were seeded in D35 dishes and exposed to MPP or LPS for indicated times. For detecting the proteins in the whole cells, cells were lysed in sample buffer [50 mM Tris-HCl (pH 6.8), 2% SDS, 10% glycerol, 0.05 g bromophenol blue, and 10 mL *β*-mercaptoethanol]. For detecting the proteins in the nucleus, nuclear extracts were prepared using a nuclear protein extraction kit (Blkw Biotech, China) according to the manufacturer's instructions. The total cell lysate or the nuclear extract was separated by SDS-PAGE and electrophoretically transferred into a nitrocellulose membrane (Millipore, USA). Then, the membrane was blocked with 5% skim milk in PBST for 1 h at room temperature and incubated with antibodies against primary antibodies at 4°C overnight. After rinsing, membranes were incubated with horseradish peroxidase- (HRP-) conjugated secondary antibody for 1 h at room temperature. The protein bands were detected by ECL reagents using ChemiDoc XRS+ system (Bio-Rad, USA). To measure the half-life of Nrf2, Beas-2B cells were left untreated or treated with MPP for 4 h and then exposed to 50 *μ*M of protein synthesis inhibitor cycloheximide (CHX). After being harvested at the indicated time, total cell lysates were subjected to immunoblot analysis.

### 2.12. Glutathione (GSH) Assay

Intracellular reduced glutathione (GSH) concentration was measured using the reduced glutathione assay kit (Jiancheng Bioengineering Institute, Nanjing, China). Beas-2B cells were seeded in D60 dishes and were treated with indicated doses of MPP for 24 h. The following procedures were carried out according to the manufacturer's instructions.

### 2.13. Reactive Oxygen Species (ROS) Detection

The effects of MPP on As(III)-induced ROS production in Beas-2B cells were determined by ROS kits (Nanjing KeyGen Biotech, China). Beas-2B cells were incubated with the indicated doses of MPP for 8 h and then treated with 5 *μ*M As(III) for 10 h. DCFH-DA (10 *μ*M) was added for additional 30 min according to the manufacturer's protocols. Cells were washed with PBS for three times and photographed using an Olympus BX53 + DP73 fluorescence imaging system (Tokyo, Japan).

### 2.14. Acridine Orange (AO)/Ethidium Bromide (EB) Staining

Beas-2B cells were seeded in D35 and preincubated with or without indicated doses of MPP for 8 h. After incubation with 5 *μ*M As(III) for 12 h, cells were washed with PBS and stained with AO/EB according to the manufacturer's instructions (Nanjing KeyGen Biotech, China). Then, cells were photographed using an Olympus 1X71 fluorescence imaging system (Tokyo, Japan).

### 2.15. Enzyme-Linked Immunosorbent Assay

The inhibitory effect of MPP against the cytokine production in the LPS-stimulated RAW 264.7 cells was determined by ELISA kit (Proteintech Group, USA). RAW 264.7 cells were seeded in a 24-well plate and were treated with indicated doses of MPP and LPS for 16 h. The following procedures were carried out according to the manufacturer's instructions.

### 2.16. Statistical Analysis

One-way analysis of variance (ANOVA) and post hoc multiple-comparison Bonferroni test were used to determine the significant difference between two groups. Results are presented as the mean ± SD. *p* < 0.05 was considered to be significant.

## 3. Results

### 3.1. Purification and Identification of Chemical Ingredients

**Figure 1:**
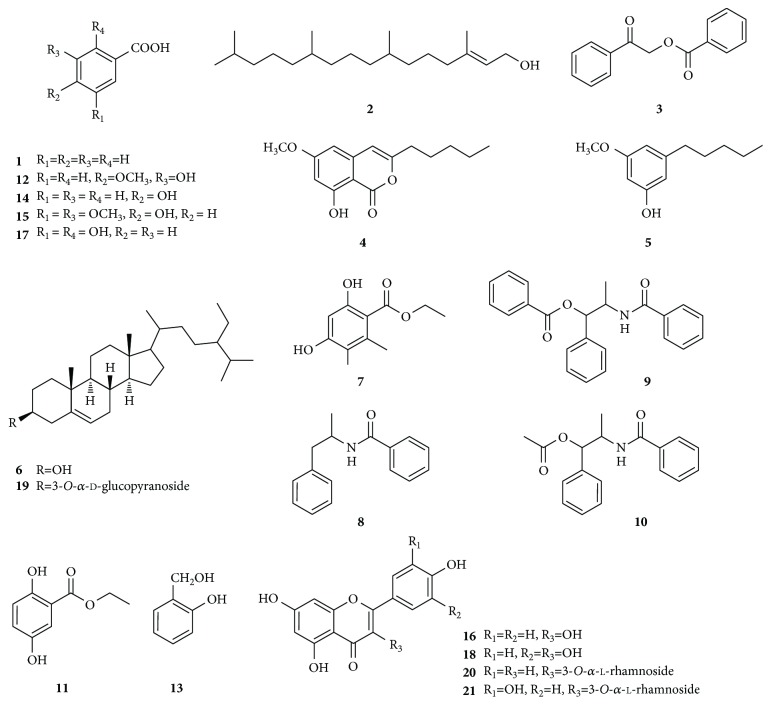
Chemical structures of the purified ingredients.

A systematic phytochemical investigation of aerial parts of *Litsea garrettii* was carried out to purify ingredients using silica gel CC, Sephadex LH-20 CC, and semipreparative HPLC. The EtOH extract was partitioned sequentially by petroleum ether, EtOAc, and *n*-butanol. Repeated chromatography of the petroleum ether and EtOAc extracts led to the isolation of twenty-one known ingredients (Figure 1). Their structures were identified to be benzoic acid (**1**) [19], phytol (**2**) [20], 1-(2-phenylcarbonyloxy acetyl)benzene (**3**) [21], 8-hydroxy-6-methoxy-3-pentylisocoumarin (**4**) [22], 3-methoxy-5-pentyl-phenol (**MPP**, **5**) [23], sitosterol (**6**) [24], ethyl orsellinate (**7**) [25], *N*-(2-phenylethyl)benzamide (**8**) [26], *N*-[2-(benzoyloxy)-1-methyl-2-phenylethyl]benzamide (**9**) [27], *N*-[2-(acetyloxy)-1-methyl-2-phenylethyl]benzamide (**10**) [28], 2,5-dihydroxybenzoic acid methyl ester (**11**) [24], isovanillic acid (**12**) [29], salicyl alcohol (**13**) [19], 4-hydroxybenzoic acid (**14**) [30], syringic acid (**15**) [29], kaempferol (**16**) [31], gentisic acid (**17**) [32], quercetin (**18**) [31], daucosterol (**19**) [24], kaempferol-3-*O*-*α*-l-rhamnoside (**20**) [33], and quercetin-3-*O*-*α*-l-rhamnoside (**21**) [33], by comparison of their NMR and MS data with those reported in the literature.

### 3.2. Identification of Bioactive Ingredients with Inducing Effect on QR

**Figure 2:**
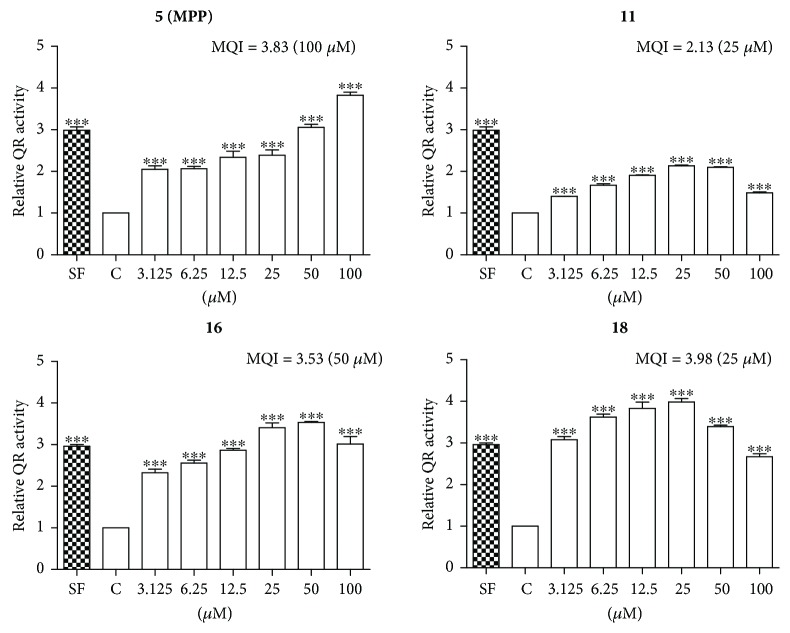
QR including activities of purified ingredients in hepa 1c1c7 cells. Cells were incubated with indicated doses of ingredients for 24 h, and then QR-inducing activity was measured. SF (2.0 *μ*M) was used as a positive control. Values were presented as mean ± SD (*n* = 3). ^∗∗∗^*p* < 0.0001, treated versus control. C: control.

The hepa 1c1c7 murine hepatoma cell line was used for screening constituents with potential inhibitory effect against oxidative stress, because of its high QR reactivity and sensitivity [17]. In this established assay, we normalized the data by setting the untreated control group as 1, and then the QR-inducing effect of tested ingredients was represented by the value of MQI (the maximum folds of QR-inducing activity compared to those of the control group). SF as a positive control demonstrated an approximately 3-fold induction at 2.0 *μ*M. The level of QR-inducing activity was ranked according to the following criteria: strong (MQI ≥ 2.5), moderate (2.5 > MQI ≥ 2), and inactive (MQI < 2). As shown in Figure 2, ingredients **5** (MQI = 3.83 at 100 *μ*M), **16** (MQI = 3.53 at 50 *μ*M), and **18** (MQI = 3.98 at 25 *μ*M) strongly enhanced QR activity in heap 1c1c7 cells, and **11** displayed moderate QR activity with a MQI value of 2.13 at 25 *μ*M. Miscellaneous tested ingredients (**1**−**4**, **7**−**15**, **17**, and **19**−**21**) are inactive in the QR-inducing effect.

### 3.3. Identification of Bioactive Ingredients with Inhibitory Effect on NO Production

**Figure 3:**
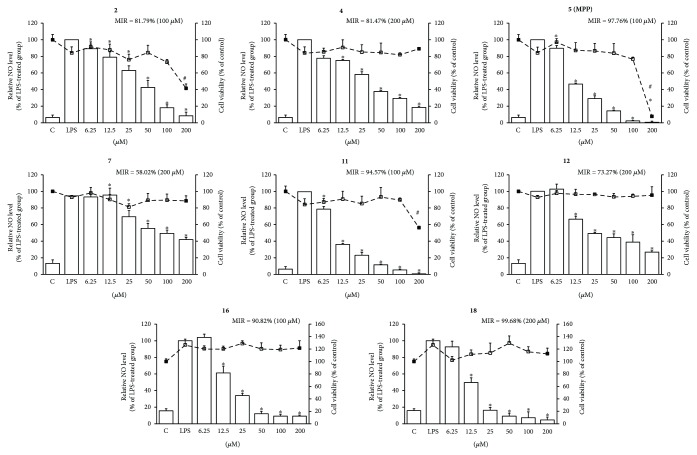
Inhibitory effect of purified ingredients on NO production in RAW 264.7 cells. Cells were treated with indicated concentrations of ingredients along with LPS (1 *μ*g/mL) for 24 h, and then the accumulation of nitrite from the supernatants was evaluated by Griess reagent. Didox was used as a positive control and possessed an inhibitory rate of about 70% at 100 *μ*M. Values were presented as mean ± SD (*n* = 3). ^∗^*p* < 0.05, relative NO level; ^#^*p* < 0.05, cell viability, treated versus LPS group. Column, relative NO level; dot, cell viability. C: control.

To address the inhibitory effect of purified constituents against inflammatory response, we used a cell-based high-throughput screening bioassay via detection of NO level in LPS-stimulated RAW 264.7 macrophages. We have simultaneously analyzed the cell viability after treatment of the purified constituents to confirm that the decrease of NO production was not attributed to the inhibition of cell proliferation. The maximum inhibition rate (MIR) of NO production under the nontoxic tested dose, which was calculated by comparing the decreased NO concentration in treated group with that in LPS-stimulated group, was used for the evaluation of anti-inflammatory potency. The inhibitory potency of NO production was ranked according to the following criteria: strong (MIR ≥ 80%), moderate (80% > MIR ≥ 50%), and inactive (MIR < 50%). As summarized in Figure 3, ingredients **2**, **4**, **5**, **11**, **16**, and **18** strongly inhibited the LPS-induced NO production in RAW 264.7 cells (MIRs ≥ 80%), while **7** and **12** exhibited moderate inhibitory effects of NO production (80% > MIR ≥ 50%). Among them, MPP (**5**) is the most potent one with a MIR of 97.76% at 100 *μ*M.

The above results indicated that 3-methoxy-5-pentyl-phenol (**MPP**, **5**), 5-hydroxyethyl salicylate (**11**), kaempferol (**16**), and quercetin (**18**) demonstrated dual potential inhibitions on oxidative stress and inflammatory response. Next, we selected MPP (**5**) for further study to investigate its potential as a preventive agent against oxidative insult and inflammatory response. The reasons for this selection are as follows: (i) MPP powerfully enhanced the induction of QR activity (MQI = 3.83) and significantly repressed the production of NO (MIR = 97.76%), with a low toxicity (minimum inhibition dose > 100 *μ*M); (ii) MPP is a novel molecule with dual antioxidant and anti-inflammatory effects, which have not been reported previously; and (iii) the capability of **11**, **16**, and **18** on inhibitions of oxidative stress and inflammation has been well-established, which lack novelty. Thus, a detailed investigation concerning the mechanism of inhibiting oxidative stress and inflammation, as well as their potential as preventive agents against oxidative insult and inflammatory response, has been performed.

### 3.4. MPP Activates Nrf2 Signaling Pathway in Human Lung Epithelial Cells

**Figure 4:**
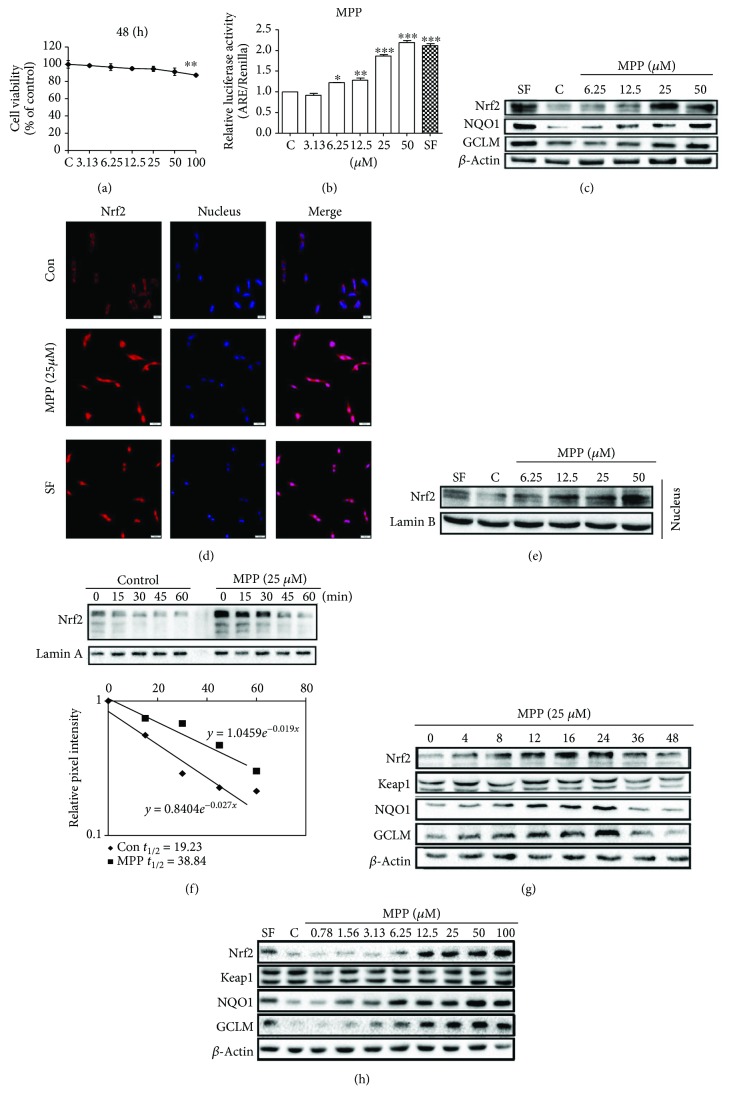
MPP activates Nrf2 signaling pathway in human lung epithelial Beas-2B cells. (a) MPP had no cytotoxicity up to 50 *μ*M. Cells were treated with indicated doses of MPP for 48 h, and then cell viability was determined using MTT assay. (b) MPP induced the ARE-dependent luciferase activity in a dose-dependent manner. Cells were transfected with ARE-firefly luciferase and TK-Renilla luciferase plasmids and then treated with the indicated doses of MPP or SF (5.0 *μ*M) for 16 h. (c) MPP dose dependently induced the protein levels of Nrf2 and its downstream genes. Cells were treated with or without indicated doses of MPP or SF (5.0 *μ*M) for 16 h, and then the total cell lysates were subjected to immunoblot analysis. (d and e) MPP induced the nuclear translocation of Nrf2. For (d), cells were treated with or without MPP (25 *μ*M) or SF (5 *μ*M) for 8 h and then subjected to indirect fluorescence staining. For (e), cells were treated with SF (5 *μ*M) and SF (5 *μ*M) and indicated doses of MPP for 8 h, and then the nuclear extracts were collected and subjected to immunoblot analysis. (f) MPP increased the half-life of Nrf2. Cells were left untreated or treated with MPP (25 *μ*M) for 4 h. Cycloheximide (50 *μ*M) was added to block protein synthesis. Cells were harvested at the indicated time points, and then total cell lysates were subjected to immunoblot analysis. (g) MPP induced Nrf2, NQO1, and GCLM but had no effect on Keap1 in the time course study. Cells were treated with MPP (25 *μ*M) for the indicated times, and then the protein levels were measured by immunoblot analysis. (h) MPP induced Nrf2, NQO1, and GCLM in a dose-dependent manner but had no effect on Keap1. Cells were treated with indicated doses of MPP for 16 h, and then the protein levels were measured by immunoblot analysis. Values were presented as mean ± SD (*n* = 3). ^∗^*p* < 0.05, ^∗∗^*p* < 0.01, ^∗∗∗^*p* < 0.0001, treated versus control; C: control.

The normal human lung epithelial Beas-2B cell line was used to evaluate the capacity of MPP on activation of Nrf2-mediated antioxidant response. We firstly tested the toxicity of MPP in Beas-2B cells to determine the treatment doses. As shown in Figure 4(a), there was no evident toxicity below 50 *μ*M for MPP. Therefore, the doses ≤ 50 *μ*M were chosen for subsequent bioassay tests. Next, we performed a Dual-Luciferase Reporter gene assay to test Nrf2 induction by MPP in Beas-2B cells. MPP dose dependently increased the ARE-luciferase activity with a maximum 2-fold induction at a dose of 50 *μ*M, compared to the untreated group (Figure 4(b)). Similarly, the protein levels of Nrf2 and its downstream genes, NQO1 and GCLM, dose dependently upregulated after exposure of cells to MPP for 16 h (Figure 4(c)). When cells are exposed to inducers, Nrf2 escapes Keap1-mediated degradation and translocates into the nucleus to regulate the transcription of a series of cytoprotective genes (e.g., NQO1 and GCLM). Thus, an indirect immunofluorescence assay and an immunoblot analysis were performed to investigate whether MPP could induce Nrf2 nuclear translocation. Not surprisingly, compared with those in the untreated control group, Nrf2 proteins mainly accumulated in the nucleus upon exposure to the MPP (25 *μ*M) (Figure 4(d)), and the protein level of Nrf2 in the nucleus dose dependently increased (Figure 4(e)). Additionally, the effect of MPP on Nrf2 stabilization was investigated by detecting the half-life of Nrf2. As shown in Figure 4(f), the half-life of Nrf2 was about 19.23 min in the untreated cells and increased to 38.84 min in response to the MPP treatment. A time course study of MPP (25 *μ*M) demonstrated that the protein levels of Nrf2, NQO1, and GCLM increased as early as 4 h, reached the highest level at 24 h, and gradually returned to basal levels by 48 h (Figure 4(g)). There was no significant change in Keap1 protein levels. Finally, we determined the protein levels of Nrf2, Keap1, NQO1, and GCLM after exposure of cells to MPP for 16 h under the doses ranging from 0.78 to 100 *μ*M (Figure 4(h)). Evident upregulation of Nrf2 protein was observed in cells treated by MPP at 6.25 *μ*M, while the downstream genes, NQO1 and GCLM, were induced by MPP at 1.56 *μ*M. All of protein levels of these three genes reached their climax at 50 *μ*M. Again, no changes in Keap1 protein levels were observed. Taken together, these data definitely suggested that MPP activated Nrf2 signaling pathway through enhancing the nuclear translocation, stabilizing Nrf2 protein, and upregulating Nrf2 at the protein level in normal human lung epithelial Beas-2B cells.

### 3.5. MPP Protects Human Lung Epithelial Cells against As(III)-Induced Oxidative Insult

**Figure 5:**
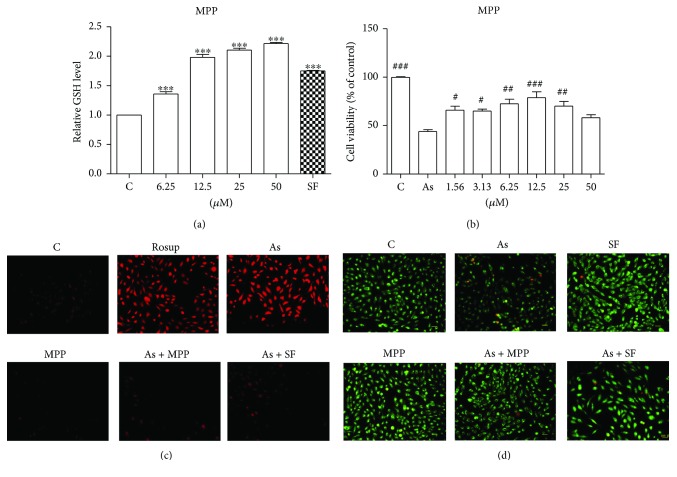
MPP protects human lung epithelial Beas-2B cells against As(III)-induced oxidative insults. (a) MPP enhanced intracellular GSH levels. Cells were exposed to the indicated doses of MPP or SF (5 *μ*M) for 24 h, and the induced GSH level was assessed using GSH detection kit. (b) MPP prevented Beas-2B cells against As(III)-induced cell death. Cells were pretreated with indicated doses of MPP for 6 h and cotreated with 5 *μ*M As(III) for 24 h. Then the cell viability was measured by MTT assay. (c) MPP inhibited the As(III)-stimulated ROS production. Cells were pretreated with or without MPP (25 *μ*M) or SF (5 *μ*M) for 6 h and then cotreated with 5 *μ*M As(III) for another 12 h. The level of ROS was evaluated using ROS detection kit. (d) MPP inhibited AS(III)-induced cell apoptosis. Cells were pretreated with or without MPP (25 *μ*M) or SF (5 *μ*M) for 6 h and cotreated with 5 *μ*M As(III) for 24 h, and then the cell viability was measured by AO/EB assay. Values were presented as mean ± SD (*n* = 3). ^∗∗∗^*p* < 0.0001, treated versus control; ^#^*p* < 0.05, ^##^*p* < 0.01, ^###^*p* < 0.0001, treated versus As. C: control.

To detect the feasibility of using MPP as a preventive agent against toxicant-induced lung tissue insult, we established an As(III)-induced cytotoxicity model *in vitro* for further investigation. Firstly, we measured intracellular glutathione (GSH) levels after MPP treatment. As depicted in Figure 5(a), MPP dose dependently increased the reduced GSH level. Then, we tested the effectiveness of MPP in protecting Beas-2B cells against As(III)-induced cell death. Pretreatment with indicated doses of MPP for 6 h evidently inhibited 5 *μ*M As(III)-induced cell death, and pretreatment with 12.5 *μ*M MPP offered the best protection (Figure 5(b)). Because As(III)-induced toxicity originated from increased ROS level [34], we determined the intracellular ROS level. Pretreatment with MPP (12.5 *μ*M) inhibited As(III)-induced increase of ROS level (Figure 5(c)). Finally, we determined the effect of MPP on As(III)-induced apoptotic cell death. AO/EB staining indicated that 5 *μ*M As(III) treatment increased the number of apoptotic cells, whereas pretreatment with MPP (12.5 *μ*M) inhibited the increase of apoptotic cell number (Figure 5(d)). Collectively, these data suggested that MPP enhanced intracellular redox capacity and protected lung epithelial cells against As(III)-induced oxidative insult.

### 3.6. MPP Inhibits LPS-Induced Inflammatory Response in RAW 264.7 Macrophages

**Figure 6:**
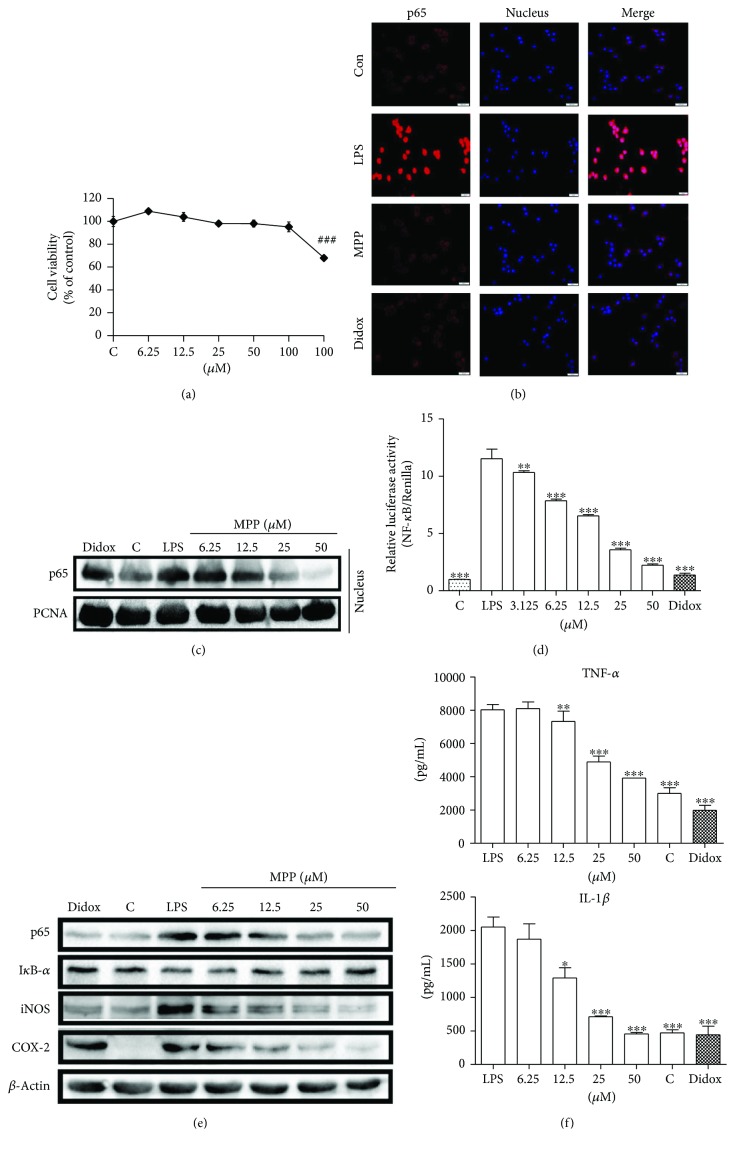
MPP inhibits LPS-induced inflammatory response in RAW 264.7 macrophages. (a) MPP had no cytotoxicity at doses ≤ 100 *μ*M. Cells were treated with indicated doses of MPP for 48 h, and the cell viability was determined by MTT assay. (b and c) MPP inhibited the LPS-induced NF-*κ*B nuclear translocation. After being pretreated with 25 *μ*M MPP for B or indicated doses of MPP for C or didox (100 *μ*M) for 1 h, cells were cotreated with LPS (1 *μ*g/mL) for 1 h and then subjected to indirect fluorescence staining and immunoblot analysis. (c) MPP inhibited the NF-*κ*B-dependent luciferase activity. After being cotransfected with NF-*κ*B firefly luciferase and TK-Renilla luciferase, cells were pretreated with MPP (25 *μ*M) or didox (100 *μ*M) for 1 h and cotreated with LPS (1 *μ*g/mL) for 16 h. (d) MPP inhibited the LPS-stimulated activation of NF-*κ*B p65 subunit, I*κ*B-*α*, iNOS, and COX-2. Cells were cotreated with indicated doses of MPP or didox (100 *μ*M) and LPS for 16 h, and then the protein levels were measured by immunoblot analysis. (e) MPP inhibited the LPS-stimulated activation of inflammatory cytokines TNF-*α* and IL-1*β*. The concentrations of cytokines were detected using the ELISA kits. Values were presented as mean ± SD (*n* = 3). ^###^*p* < 0.0001, treated versus control; ^∗^*p* < 0.05, ^∗∗^*p* < 0.01, ^∗∗∗^*p* < 0.0001, treated versus LPS. C: control.

To evaluate the toxicity of MPP and determine the treatment doses for the subsequent RAW 264.7 cell-based experiments, a MTT assay was carried out. As depicted in Figure 6(a), no evident toxicity was observed when cells were exposed to MPP at doses below 100 *μ*M for 48 h. Thus, the doses less than 100 *μ*M for MPP were chosen for subsequent bioassay tests. When cells are exposed to LPS, NF-*κ*B escapes from the binding with I*κ*B-*α*, translocates to the nucleus, and activates the inflammation-related gene expressions. Therefore, we performed immunofluorescence assay and immunoblot analysis to detect whether MPP could revert the LPS-induced NF-*κ*B nuclear translocation. As show in Figures 6(b) and 6(c), NF-*κ*B p65 subunit accumulated in the nuclear after stimulation by LPS, while treatment with MPP and didox blocked the process of NF-*κ*B p65 subunit nuclear translocation. Next, we performed a Dual-Luciferase Reporter gene assay to confirm the inhibition of MPP on NF-*κ*B in LPS-stimulated RAW 264.7 cells. Compared with the untreated group, LPS treatment potently activated the NF-*κ*B reporter activity. MPP dose dependently inhibited LPS-stimulated increase of NF-*κ*B luciferase activity (Figure 6(d)). Similarly, the protein level of p65 decreased in a dose-dependent manner after exposure of cells to MPP for 16 h (Figure 6(e)). Since I*κ*B-*α* degradation was the key step for regulation of NF-*κ*B activation, we measured the effect of MPP on I*κ*B-*α* content. As expected, the protein level of I*κ*B-*α* decreased after LPS treatment, which could be dose dependently reverted by MPP (Figure 6(e)). Finally, we determined the effects of MPP on NF-*κ*B-regulated inflammatory proteins iNOS and COX-2, and cytokines TNF-*α* and IL-1*β*. MPP inhibited the LPS-stimulated upregulation of iNOS and COX-2 protein levels and suppressed the LPS-stimulated production of TNF-*α* and IL-1*β* in a dose-dependent manner (Figure 6(f)). Taken together, these data demonstrated that MPP inhibited LPS-induced inflammatory response through blocking the NF-*κ*B activation.

## 4. Discussion

*Litsea garrettii* Gamble [synonym *L. martabanica* (Kurz) Hook. f.] belongs to the family of Lauraceae and mainly occurs in Thailand, Myanmar, and the south of China [18]. However, only a few findings on its phytochemistry and pharmacology have so far been reported. In the present study, twenty-one ingredients were isolated from this plant for the first time, covering eight benzoic acid derivatives (**1**, **3**, **7**, **11**, **12**, **14**, **15**, and **17**), one aliphatic compound (**2**), one coumarin (**4**), two phenol derivatives (**5** and **13**), three benzamides (**8**−**10**), two steroids (**6** and **19**), and four flavonoids (**16**, **18**, **20**, and **21**) (Figure 1). To the best of our knowledge, this is the first investigation on the phytochemical aspect of *L. garrettii*, and thus, all of the ingredients were firstly purified from this plant, which definitely enriched its chemical diversity.

We have previously discovered that the EtOH extract of *L. garrettii* activated Nrf2 signaling pathway and protected human bronchial epithelial cells against H_2_O_2_-induced oxidative insult [16]. Through analyzing the QR-inducing effects of purified ingredients, MPP (**5**), benzoic acid analogue (**11**), and flavonoids (**16** and **18**) were suggested to be the bioactive constituents supporting the cytoprotection against oxidative insult (Figure 2). The anti-inflammatory effects of purified ingredients were tested for the inhibition against the production of NO, which is a marker for inflammatory response [35]. Eight ingredients (**2**, **4**, **5**, **7**, **11**, **12**, **16**, and **18**) inhibited the production of NO (Figure 3). Importantly, MPP (**5**) and ingredients (**11**, **16**, and **18**) possessed dual biological functions on the induction of QR and inhibition of NO production. Of the four active ingredients, the benzoic acid analogue (**11**) and flavonoids (**16** and **18**) are previously identified inhibitors of oxidative stress and/or inflammatory response [36–40]. Compared with those on ingredients **11**, **16**, and **18**, few findings on the antioxidant and anti-inflammatory effects of MPP have been published. It has been reported that MPP displayed inhibitions against 5-lipoxygenase [41], *α*-glucosidase [42], and *Mycobacterium tuberculosis* H37Rv *in vitro* [43].

A growing body of evidences indicated that Nrf2 played a central role in maintaining the intracellular redox homeostasis to counteract exogenous oxidants- and toxicants-induced oxidative damages [10, 44, 45]. Hence, *in vitro* experiments using human lung epithelial Beas-2B cells were carried out to investigate the mechanism of MPP on inhibiting oxidative insult targeting Nrf2 signaling pathway. Our results indicated that MPP significantly induced Nrf2 and its downstream genes, NQO1 and GCLM, and enhanced the nuclear translocation and stabilization of Nrf2 in Beas-2B cells (Figure 4). Meanwhile, time course study indicated that MPP-mediated Nrf2 activation was intermittent in Beas-2B cells (Figure 4(g)), a similar manner with that of chemopreventive agents (e.g., SF) [34]. These data definitely supported that MPP was a canonical Nrf2 activator.

The widespread environmental contaminant, arsenic, has been defined as human toxicant. Epidemiological studies indicated that exposure to arsenic caused many health disorders, including cancer, diabetes, and chronic inflammation [44]. Arsenic-induced cytotoxicity is closely related to its capability of disturbing cellular redox homeostasis via depleting reduced GSH and generating ROS [46, 47]. Therefore, we established an arsenic-based cell model *in vitro* to test the capability of MPP on the inhibition of oxidative insult. Treatment with MPP increased cellular reduced GSH level and inhibited As(III)-induced ROS production (Figures 5(a) and 5(c)). The upregulation of reduced GSH by MPP was consistent with its action on the increased protein level of GCLM, a key enzyme for GSH synthesis [48]. Accordingly, downregulation of ROS production by MPP inhibited As(III)-induced death and cell apoptosis (Figures 5(b) and 5(d)). These observations definitely verified the preventive effect of MPP against As(III)-induced oxidative insult.

LPS, an outer membrane component of Gram-negative bacteria, could directly stimulate NF-*κ*B-mediated inflammatory response and activate the production of inflammatory mediators [49]. As depicted in Figure 6(a), exposure of cells to LPS evidently activated NF-*κ*B p65 translocation and upregulated the levels of COX-2, iNOS, IL-1*β*, and TNF-*α*, supporting the effectiveness of LPS-stimulated cell model in RAW 264.7 macrophages. NF-*κ*B is a key regulator of inflammatory response in stimulated macrophages [50]. The association of NF-*κ*B p65/p50 is localized in the cytoplasm in an inactive form associated with I*κ*B. In response to LPS, I*κ*B is degraded via phosphorylation, which gives rise to the translocation of NF-*κ*B and induction of transcription of proinflammatory genes [14]. Treatment with MPP significantly inhibited the LPS-stimulated I*κ*B-*α* degradation and NF-*κ*B p65 activation and translocation (Figures 6(b)–6(e)). The key inflammatory mediator, NO, is able to mediate the functions of inflammatory cells, including macrophages, T lymphocytes, and monocytes, and promote the production of cytokine and matrix metalloproteinase to enhance the inflammatory response [51, 52]. Our data indicated that MPP inhibited the LPS-induced upregulation of NO, as well as iNOS, a key enzyme for NO synthesis (Figures 3 and 6(e)). COX-2 catalyzes arachidonic acid to synthesize prostanoids (e.g., PGE2), which facilitates the influx of neutrophils, and macrophages from bloodstream to swelling and edema at infection or injury [53]. Besides iNOS and COX-2, LPS-stimulated macrophages produce proinflammatory cytokines (e.g., TNF-*α* and IL-1*β*) [54]. TNF-*α* is a primary endogenous mediator of inflammatory response and contributes to the sustentation of inflammation through activating T lymphocytes and macrophages [55, 56]. Similarly, IL-1*β* is an inflammatory mediator produced by stimulated macrophages and involved in many inflammatory processes, for instance, activation of COX-2 [55]. Our data indicated that MPP markedly downregulated the protein level of COX-2 and reduced the production of TNF-*α* and IL-1*β* in LPS-induced RAW 264.7 cells (Figures 6(e) and 6(f)). Collectively, our results implied the prevention of MPP against LPS-induced inflammatory response.

## 5. Conclusion

In summary, twenty-one ingredients have been isolated from *Litsea garrettii* for the first time, which enriched the chemical diversity of this plant. MPP (**5**), 2,5-dihydroxybenzoic acid methyl ester (**11**), kaempferol (**16**), and quercetin (**18**) possessed potential dual inhibitions against oxidative stress and inflammation. MPP (**5**) is identified to be an activator of Nrf2 and an inhibitor of NF-*κ*B and protects cells against As(III)-induced oxidative insult and LPS-induced inflammatory response. These data implied the potential uses of MPP as a preventive agent against oxidative stress- and inflammation-related diseases, such as COPD, cancer, and diabetes mellitus.

## Abbreviations

AO: Acridine orange
ARE: Antioxidant response element
As(III): Sodium arsenite
CC: Column chromatography
CHX: Cycloheximide
COPD: Chronic obstructive pulmonary disease
COX-2: Cyclooxygenase-2
DAPI: 4′,6-Diamidino-2-phenylindole
EB: Ethidium bromide
GCLM: Glutamate-cysteine ligase regulatory subunit
iNOS: Inducible nitric oxide synthase
I*κ*B: Inhibitors of *κ*B
IL-1*β*: Interleukin-1*β*
Keap1: Kelch-like ECH-associated protein 1
MTT: 3-(4,5-Dimethylthiazol-2-yl)-2,5-diphenyltetrazoliumbromide
NF-*κ*B: Nuclear factor *κ*B
NO: Nitric oxide
NQO1: NAD(P)H:quinone oxidoreductase
Nrf2: Nuclear factor erythroid 2-related factor 2
QR: NAD(P)H:quinone reductase
ROS: Reactive oxygen species
SF: Sulforaphane
TNF-*α*: Tumor necrosis factor *α*.
